# Mothers’ Perceptions of the Internet and Social Media as Sources of Parenting and Health Information: Qualitative Study

**DOI:** 10.2196/14289

**Published:** 2019-07-09

**Authors:** Rachel Y Moon, Anita Mathews, Rosalind Oden, Rebecca Carlin

**Affiliations:** 1 University of Virginia Charlottesville, VA United States; 2 Children's National Health System Washington, DC United States

**Keywords:** internet, parenting, social media, focus groups

## Abstract

**Background:**

Traditionally, guidance and support to new parents have come from family, friends, and health care providers. However, the internet and social media are growing sources of guidance and support for parents. Little is known about how the internet and social media are used by parents of young infants and specifically about parental perceptions of the internet and social media as sources of parenting and infant health information.

**Objective:**

The aim of this study was to explore, using qualitative methods, parental perceptions of the advantages and disadvantages of the internet and social media as sources of parenting and health information regarding their infant.

**Methods:**

A total of 28 mothers participated in focus groups or individual interviews. Probing questions concerning parenting and health information sources were asked. Themes were developed in an iterative manner from coded data.

**Results:**

The central themes were (1) reasons that mothers turn to the internet for parenting and health information, (2) cautionary advice about the internet, and (3) reasons that mothers turn to social media for parenting and health information. Mothers appreciated the ability to gather unlimited information and multiple opinions quickly and anonymously, but recognized the need to use reputable sources of information. Mothers also appreciated the immediacy of affirmation, support, and tailored information available through social media.

**Conclusions:**

The internet and social media are rapidly becoming important and trusted sources of parenting and health information that mothers turn to when making infant care decisions.

## Introduction

Most adults find parenting to be an entirely new experience for which they have little background knowledge. It can easily become overwhelming, and parents need guidance and support. Traditionally, guidance and support have come from family, friends, and health care providers [1-4]. However, just as the internet is becoming a growing source of health information in general, with 59% of US adults seeking health information on the internet [5], it is also a growing source of guidance and support for parents [6,7].

In addition, the recent advent of social media (defined as forms of electronic communication through which one can share information, ideas, and personal messages with others online [8]), including online social networks (eg, Facebook), email listservs, blogs, and mobile phone apps, has introduced new channels through which one can seek information and the opinions of others. Parents, in particular, have found these interactive forums helpful. The majority of US parents who use social media state that it provides useful parenting information, and almost half have received support on social media regarding a parenting issue in the past month [9]. Mothers of young infants find social networking sites to be important sources of social support [10].

Our previous research has found that mothers are more consistently the primary decision maker pertaining to the infant [1]. In addition, women are more likely to seek advice and help from multiple sources, including social media [9], whereas men are more likely to depend almost solely upon their spouses [11,12]. Little is known about how the internet and social media are used by mothers of young infants as sources of parenting and health information. Thus, we conducted a qualitative study to explore maternal perceptions of the internet and social media as parenting and health information sources.

## Methods

### Recruitment

Mothers of healthy term infants <6 months of age were recruited to participate in a larger quantitative survey about their personal social networks if they were English speaking, African American (AA) or Caucasian, aged >18 years, a primary caregiver for the infant, and lived in the metropolitan Washington DC area. Each mother signed a written informed consent upon enrollment. From this sample, we selected a subsample [13] to participate in focus groups or individual interviews. We purposefully chose both AA and Caucasian mothers with different educational and socioeconomic levels to assure a wide range of attitudes and opinions. Socioeconomic levels were dichotomized (lower and higher) based on enrollment in public health insurance (Medicaid or the equivalent) and eligibility for the Special Supplemental Nutrition Program for Women, Infants, and Children. These proxies were used because eligibility for both programs are income based and because eligibility is easily verifiable.

#### Data Collection

We conducted all interviews between July 2016 and January 2018. We conducted both focus group interviews and individual in-depth semistructured interviews to accommodate mothers’ schedules. Mothers who could not participate in focus groups were offered individual interviews. Focus groups were stratified by race and parity (primiparous or multiparous) to maximize homogeneity of each group’s participants, as this can result in increased willingness to share thoughts and opinions [14]. Interview questions were formulated by all authors in group meetings; the same interview guide was used for both interview formats. Questions were modified iteratively based on data collected in previous interviews. Trained facilitators (RO and AM) conducted all the interviews, asking broad, open-ended questions (eg, “What sources, other than your family and friends, do you use for advice?”) followed by more specific, probing questions (eg, “Why that source, in particular?”) to clarify responses. Focus group and individual interviews averaged 2 hours and 90 min, respectively, in duration. Each participant received a US $75 gift card for their time. This study was approved by the institutional review boards of Children’s National Medical Center and the University of Virginia.

### Data Analysis

All interviews were video- and audio-recorded and transcribed by a Health Insurance Portability and Accountability Act–compliant transcription company, after which one author (RO) simultaneously reviewed the video- and audio-recordings and transcript of each interview for accuracy. Any disagreements about the transcription were resolved by consensus after additional authors listened to the recordings. This multistep process was used to maximize accuracy and eliminate bias from the transcription process. Using standard qualitative analytic techniques and a grounded theory approach, transcripts were analyzed line-by-line by the 4 authors, all of whom have previous experience with [1,15-18] or training in qualitative analysis. Qualitative analysis software (NVivo 11 plus [19]) was used to organize, sort, and code the data (quotations). Themes were developed and revised iteratively, as patterns within data emerged [20]. Authors, in regular meetings, discussed emerging themes and patterns in the data and reached a consensus on the major themes. Individual and focus group interviews were analyzed separately, and emerging themes were compared. To increase rigor, concurrent triangulation or use of multiple sources for verification of findings [21] of the focus group interviews and the individual interviews was used to corroborate findings [22]. In addition, we confirmed the findings by peer review and feedback during presentations to child health professionals, pediatric researchers, and community members.

## Results

### Demographics

A total of 8 focus groups and 2 individual interviews with 28 mothers (26 participated in focus groups [median 3.5 participants, range 2-6 participants]; 2 participated in individual interviews) were conducted, and thematic saturation was reached. At the time of the interview, mothers had a mean age of 30.4 years (range 20-44 years), 71.4% of the mothers were AA (which reflects the racial distribution in the larger sample and is representative of the general population in Washington DC), and slightly over half of the mothers were primiparous; 61% of women were married, and 64.3% of infants were male (see Table 1).

**Table 1:** Characteristics of participants (N=28).

Characteristics		Statistics
Age (years), mean (range)		30.4 (20-44)
**Age (years), n (%)**		
	18-24	5 (18)
	25-29	10 (36)
	30-34	5 (18)
	≥35	8 (29)
**Race or ethnicity, n (%)**		
	Black or African American	20 (71)
	Caucasian	8 (29)
**Educational level, n (%)**		
	Did not complete high school	1 (4)
	High school graduate	6 (21)
	Some college	4 (14)
	4-year college graduate	17 (61)
**Socioeconomic status, n (%)**		
	Lower	10 (36)
	Higher	18 (64)
**Marital status, n (%)**		
	Married	17 (61)
	Never married	10 (36)
	Separated/divorced	1 (4)
**Number of children, n (%)**		
	1	15 (54)
	2 or more	13 (46)
**Infant gender, n (%)**		
	Male	18 (64)
	Female	10 (36)
Infant age (months), mean (range)		4.8 (1-11)
**Infant age (months), n (%)**		
	<3	7 (25)
	3-6	15 (54)
	>6	5 (18)

### Central Themes

Several main themes and subthemes with regard to maternal use of the internet and social media are listed in Table 2. The central themes were (1) reasons that mothers turn to the internet for parenting and health information, (2) cautionary advice about the internet, and (3) reasons that mothers turn to social media for parenting and health information. These themes, with illustrative quotes, are discussed below.

### Reasons That Mothers Turn to the Internet for Parenting and Health Information for Their Infant

Mothers reported that they often had multiple questions about their infant every day, particularly when they were new at mothering, and they appreciated that there was unlimited information at their fingertips:

[The Internet] is also good for informational purposes...there’s nothing wrong with having an endless amount of information at your disposal. What’s wrong with educating yourself on these sorts of things? And so, I believe in higher learning so I can have information at any way possible. And so, the one great thing about this is you get information in the click of a finger. I don’t see anything wrong with constantly sourcing information. And then in terms of something as important as your baby, like this is my first and only kid. I want to know, you know, and I want to sort of make the best choices and decisions that I possibly can so you aim at a healthy direction.

Mothers particularly appreciated that they could use the internet as a way to quickly *crowdsource* or gather multiple viewpoints when trying to make a decision regarding their infant. One mother compared the unlimited amount of information on the internet with her mother, who was a single source of information:

I really like gathering information, and the Internet’s kind of an unlimited way as a source of information. And you can go to lots of sources at one time, versus like, my mom is one and it’s limited.

The internet was also considered a way to obtain information anonymously, either because mothers did not want to ask questions that might have an obvious answer or because the questions were too personal to ask someone:

That blog has a lot of questions and answers. You can post things kind of anonymously so you don’t kind of feel like I’m asking a stupid question. Like you can put like, “Is it normal for babies to have a green poop?”

Also, there are some things I don’t want to ask friends that might feel more personal.

**Table 2:** Themes and subthemes about maternal use of the internet and social media.

Themes	Subthemes
Reasons that mothers turn to the internet for parenting and health information for their infant	Unlimited information available; Anonymity; Convenience; Immediate answers; Faster than information from a health care professional; Trustworthiness of the information; Up to date; Use to confirm information obtained from other sources
Cautionary advice about the internet	Wealth of information is overwhelming; Many nonreputable websites; Generalizability of the information
Reasons that mothers turn to social media for parenting and health information	Immediate affirmation and support; Honest answers; Acts as a support group; Tailored information; Trustworthiness of information

As mothers often did not want to bother a health care professional or another person with all of their questions, they found the internet to be a convenient place to obtain information, particularly information that was not urgent. They could often find that information much more quickly than they would have if they had contacted a health care provider:

And then some things, there might be medical things. I must have called my doctor’s office like twice a week, every week, for like nine months. They go, “Hi again.” At some point I’m like, I'm wearing out my poor nurses.

Because sometimes [going to the internet] is easy. If you’re just sitting there and it’s a question that’s not...life or death...like, what colors should babies’ poop be? Like, you’re going to get the right answer and the Internet’s going to get that right. I don’t think that somebody’s going to be wrong about it. So, for stuff like that I feel like there’s no reason to bother somebody else with that kind of question.

Mothers described the information on websites as generally trustworthy. They trusted that it was more current than the information that they received from trusted family members and friends:

I read some of them on the Internet if there are questions about behavioral changes or developmental stages, because I think a lot of older resources like my parents, maybe they’ve forgotten what that stuff is like.

Thus, they tended to use the internet to confirm the information obtained from other sources and for reassurance that their child was normal:

[I go to the internet] just for confirmation. Just to see... There may be something else. There may be something else that my mom doesn’t know.

Yeah. So, [the internet] just gives me a heads up on what things to look out for and if there’s any concerns, like if my baby isn’t smiling on a social level or hasn’t at least rolled over by now, so I know, okay, I need to consult with my social network regarding what I’m seeing.

### Cautionary Advice About the Internet

When mothers described their information sources on the internet, they cautioned each other to be careful about using only reputable websites for information. Websites such as Baby Center and WebMD, and search engines such as Google, are among those cited by mothers as being reputable sources:

If we have a question about something, [then I can] Google, “why does my baby whatever?” And then I’ll sort of look at what comes up, and I’ll go to what I feel is the most reputable source on there and sort of read what they have to say about it...There’s usually somebody out there who has something to say about the question. It’s amazing what you can Google and somebody’s asked it before.

I will say, do your research and just find...for instance, Google, WebMD, Baby Center, kind of read what people are saying, what the doctors are saying online. And then find a reliable source through the Internet if you don't have someone physically there. If it gave me good advice before, I’ll go back to it again.

In addition, mothers also described that the wealth of information could also become overwhelming, particularly when there were multiple differing viewpoints:

Everyone has a million and one opinions and every[one] has a different expert advice.

Some mothers found the information on the internet to be so general as to be unhelpful to their specific situation. Mothers wanted the websites to be tailored to their situation and, occasionally, to their own race or ethnicity, to be assured about the websites’ reliability:

I think especially with having a little black baby...The medical field...it’s geared toward other folks and their kids...If he has cradle cap, the pictures I’m seeing are not little babies that look like my baby.

### Maternal Perceptions of Social Media

With regard to social media, such as social network sites, email listservs, blogs, and mobile phone apps, mothers frequently used these as sources of parenting and health information and were generally extremely positive about the information that they found on these sites. They appreciated the immediacy of the affirmation and support that they found in these forums. Mothers described situations in which they asked a question about negative child-rearing experiences and received what they perceived to be honest answers:

People are open about [difficult experiences], but you have to ask them about it. Nobody wants to talk about like, “Oh, you have a beautiful baby. Wait until this happens, because it’s terrible.” And so, I don’t think people talk about it. But once you bring it up, people are like, “Oh, yeah. We dealt with terrible diaper rash or we dealt with bad breastfeeding,” or whatever it is...once you start talking to people, they're very willing to talk about it.

In this way, these forums often served as virtual support groups for the mothers, providing affirmation about their situation and the infant:

You like to think your baby’s unique. [But] every baby has the same issues when it comes down to it and so somebody’s had to deal with it at some point.

Many mothers used social media forums, specifically email listservs, as a way to meet other mothers and share experiences:

And over the years I’ve been using those more and more, joining more and more listservs. And then I came to find out that there were some moms’ groups, specifically in [your town]...and they have cohorts based on the week your child was born, and you meet for several weeks, and then those women become friends.

Mothers who had previous experience with child-rearing remarked on how the explosion of social media websites has changed how they ask for and receive information. These are become increasingly important as sources of information, with the information generally immediate and tailored to their situation:

People use Facebook groups a lot more than they did seven years ago. So, for example, just yesterday a woman posted on Facebook on our moms’ group, “I just had my baby four hours ago and he’s not nursing and I had a Caesarean. I know that this isn’t an emergency, but I wanted the moms to weigh in on what I should be doing...” And probably about 12 of us responded right away. And I...just gave her what had happened to me...She got an immediate moms’ [support group]; I wish I had that when I...was in the same situation...If [I] had known that there was this option of insta-moms on Facebook, that would’ve been a dream for me.

They appreciate the discussions that occur in these forums and find them relevant and trustworthy. Some mothers prefer the advice coming from social media to their health care provider’s advice:

I find that the listserv information is more reliable...we had these four-week-old babies...And a mom said that her baby was constipated and that the doctor’s advice was to give him...a laxative, but I was like, “Oh my God.” I mean, I think I felt like my heart stopped...I’m still traumatized by some poor mother following advice from a crazy pediatrician to think that you would give a baby some; it made no sense to me. So, I find if she had asked that question at the listserv, she would get better advice than following the advice of this doctor.

Mothers who used mobile phone apps in particular liked how the information on the apps was timely and tailored to their individual situation:

You can tailor [the apps and websites] to your child’s age and development, and that’s why I appreciate that. Because sometimes the other stuff, the other sources of advice, they’re just this nebulous baby advice but [not] based on what [my baby is doing].

## Discussion

### Principal Findings

The internet and social media have rapidly become important influences, impacting numerous decisions on a daily basis. Opinions expressed online by strangers are as, or potentially more, important than those of family and friends [23-26]. In a 2018 Pew survey, 11% of US adults stated that they changed their opinion because of what they saw on social media in the past year [27]. Although these statistics are not specific to health-related decision making, our findings suggest that maternal decisions related to infant care may also be strongly influenced by what mothers access on the internet and social media.

Social learning theories [28,29] suggest that behaviors and rationalizations for those behaviors are in part learned from and reinforced by others, so that one’s behavior becomes increasingly similar to that of the people to whom she/he has regular exposure. The theory of reasoned action similarly argues that volitional behavior results, in part, from subjective norms regarding a behavior, that is, one’s perception of the prevailing attitudes and beliefs regarding a behavior that are held by people whom one trusts [30-32]. Our findings suggest that the internet and social media have become as influential as family and friends in modeling behavior, establishing norms, and thus shaping parenting and health-related decision making.

Mothers appreciated the fact that the internet and social media allowed them to access unlimited information instantaneously on a 24/7 basis. They did not need to wait for the next well child visit or to call their health care professional, family member, or friend for advice. They also liked the anonymity of the internet [33], which allowed them to ask questions that they might be embarrassed to ask a person.

Mothers liked to crowdsource information, that is, to gather opinions from multiple sources [9,15]. We and others have found that mothers make decisions by instinct [34,35] and/or consensus [1,15]. If there was a general agreement about the decision, then that allowed them to move forward with that decision with more confidence. Results from this study suggest that the internet, with its access to vast numbers of opinions, provided this consensus and confidence [35]. 

Mothers believed that information on the internet is generally trustworthy, with the caveat that you have to use reliable websites and apps. Although we did not ask them to define how they determine whether a website is reliable, they commented that previous good experience with a source increased their trust in it and that they generally went back repeatedly to those websites/apps with which they had positive previous experiences. Many volunteered specific sources that they considered trustworthy, for example, WebMD and Baby Center. Although it is not technically a website, Google was a frequently cited search engine. However, parenting information found in websites that rank highly in Google search results may often be unsupported by evidence [36]. One study found that, when it came to information about infant sleep safety, government websites were the most accurate and blogs the least [36]. Therefore, the fact that no women in our sample explicitly cited concerns about information on blogs or other crowdsourcing websites, despite the fact that these sites are frequently consulted, is concerning.

Indeed, although mothers in our study cautioned that only reliable websites should be trusted, this concern was not expressed about social media. Perhaps because these Facebook groups, blogs, and listservs were geared toward parents and because entries and responses were written by those perceived by mothers to be *just like me*, mothers in our study considered the information and opinions expressed on social media as being trustworthy, perhaps even more trustworthy than those of health care professionals. Bernhardt, in a qualitative study of southwestern US parents, found that mothers highly trusted internet content written by other parents but only in specific questions [37]. Kallem found that, in a Facebook group of mothers, peer responses to mothers’ direct questions about their infants’ health generally did not contradict American Academy of Pediatrics (AAP) recommendations. However, mothers’ posts describing their infants’ sleep practices and screen time practices frequently were not consistent with AAP recommendations [38]. It is thus concerning that this information is so highly trusted. Although there is no published research specifically on the effectiveness of health care providers guiding parents to access vetted websites and more monitored social media sites, health care providers are trusted sources of health information for many parents [39,40]. Thus, parents may appreciate their guidance regarding appropriate internet sources of parenting and health information, and this guidance may in turn improve the safety of infant care practices.

### Limitations

We acknowledge that this study has several limitations. First, our study population was limited to a single geographic region, to mothers, and to those who self-identified as AA or Caucasian. Second, although qualitative research can provide an insight into a broad array of opinions, it cannot be used to determine the prevalence of any one viewpoint. Thus, although we reached thematic saturation, our results are not necessarily generalizable to fathers, other groups, or geographic regions. However, our findings are consistent with other qualitative studies [34,35,37]. Nonetheless, further study in other geographic and racial/ethnic groups will be important to determine if these perceptions and opinions are widespread.

### Conclusions

The internet and social media are becoming important sources of health information that mothers turn to when making infant care decisions, and for some mothers, these electronic resources are more trusted than family members, friends, and health care professionals. It is becoming increasingly important that parents be provided guidance about accessing trustworthy, evidence-based health information. In addition, health care providers will need to be proactive in harnessing social media to encourage healthy decisions.

## Abbreviations

AA: African American
AAP: American Academy of Pediatrics
